# Analysis of Porcine Transcriptional Response to *Salmonella enterica *serovar Choleraesuis suggests novel targets of NFkappaB are activated in the Mesenteric Lymph Node

**DOI:** 10.1186/1471-2164-9-437

**Published:** 2008-09-23

**Authors:** Yanfang Wang, Oliver P Couture, Long Qu, Jolita J Uthe, Shawn MD Bearson, Daniel Kuhar, Joan K Lunney, Dan Nettleton, Jack CM Dekkers, Christopher K Tuggle

**Affiliations:** 1 Department of Animal Science, and Center for Integrated Animal Genomics, Iowa State University, 2255 Kildee Hall, Ames, IA 50010, USA; 2 National Animal Disease Center, USDA-ARS, 2300 Dayton Road, Ames, IA 50010, USA; 3 Animal Parasitic Diseases Laboratory, ANRI, USDA-ARS, Beltsville, MD 20705, USA; 4 Department of Statistics, Iowa State University, 111B Snedecor Hall, Ames, IA 50010, USA

## Abstract

**Background:**

Specific knowledge of the molecular pathways controlling host-pathogen interactions can increase our understanding of immune response biology as well as provide targets for drug development and genetic improvement of disease resistance. Toward this end, we have characterized the porcine transcriptional response to *Salmonella enterica *serovar Choleraesuis (*S*. Choleraesuis), a *Salmonella *serovar that predominately colonizes swine, yet can cause serious infections in human patients. Affymetrix technology was used to screen for differentially expressed genes in pig mesenteric lymph nodes (MLN) responding to infection with *S*. Choleraesuis at acute (8 hours (h), 24 h and 48 h post-inoculation (pi)) and chronic stages (21 days (d) pi).

**Results:**

Analysis of variance with false discovery rate control identified 1,853 genes with significant changes in expression level (*p*-value < 0.01, *q*-value < 0.26, and fold change (FC) > 2) during infection as compared to un-inoculated control pigs. Down-regulation of translation-related genes at 8 hpi and 24 hpi implied that *S*. Choleraesuis repressed host protein translation. Genes involved in the Th1, innate immune/inflammation response and apoptosis pathways were induced significantly. However, antigen presentation/dendritic cell (DC) function pathways were not affected significantly during infection. A strong NF*κ*B-dependent response was observed, as 58 known NF*κ*B target genes were induced at 8, 24 and/or 48 hpi. Quantitative-PCR analyses confirmed the microarray data for 21 of 22 genes tested. Based on expression patterns, these target genes can be classified as an "Early" group (induced at either 8 or 24 hpi) and a "Late" group (induced only at 48 hpi). Cytokine activity or chemokine activity were enriched within the Early group genes GO annotations, while the Late group was predominantly composed of signal transduction and cell metabolism annotated genes. Regulatory motif analysis of the human orthologous promoters for both Early and Late genes revealed that 241 gene promoters were predicted to contain NF*κ*B binding sites, and that of these, 51 Early and 145 Late genes were previously not known to be NF*κ*B targets.

**Conclusion:**

Our study provides novel genome-wide transcriptional profiling data on the porcine response to *S*. Choleraesuis and expands the understanding of NF*κ*B signaling in response to *Salmonella *infection. Comparison of the magnitude and timing of porcine MLN transcriptional response to different *Salmonella *serovars, *S*. Choleraesuis and *S*. Typhimurium, clearly showed a larger but later transcriptional response to *S*. Choleraesuis. Both microarray and QPCR data provided evidence of a strong NF*κ*B-dependent host transcriptional response during *S*. Choleraesuis infection. Our data indicate that a lack of strong DC-mediated antigen presentation in the MLN may cause *S*. Choleraesuis infected pigs to develop a systemic infection, and our analysis predicts nearly 200 novel NF*κ*B target genes which may be applicable across mammalian species.

## Background

*Salmonella enterica *serovar Choleraesuis (*S*. Choleraesuis) is a narrow host range *Salmonella *serovar that predominately colonizes swine, although this serovar can also cause serious infections in human patients [1,2]. With clinical manifestations of enterocolitis, pneumonia, septicemia and hepatitis, *S*. Choleraesuis infections result in a higher mortality rate in pigs than *S*. Typhimurium, a broad host range S*almonella *serovar that typically causes only enterocolitis in swine. This highly invasive serovar is of particular concern to the swine industry since pig salmonellosis results in about $100 million in annual production losses in the United States [3]. *S*. Choleraesuis can result in higher mortality than S. Typhimurium infection in pigs. Moreover, *S*. Choleraesuis infected pigs can develop a carrier state lasting as long as 12 weeks [4]; resulting in shedding of the bacteria by carrier pigs and *Salmonella *contamination of the environment. Researchers have speculated that human systemic infections caused by *S*. Choleraesuis are acquired from pigs, as affirmed by DNA fingerprints of the *S*. Choleraesuis isolated from humans and swine [1,2,5]. Although an oral *S*. Choleraesuis vaccine (ENTERISOL^® ^SC-54) has been shown to enhance humoral and cellular immune responses [6], *S*. Choleraesuis infections can be particularly difficult to treat due to its resistance to multiple antimicrobial agents. Thus *S*. Choleraesuis is both a swine industry and public health problem.

While several serovars such as *S*. Typhimurium have been extensively studied, a relatively limited number of experiments on the *S*. Choleraesuis serovar have been published using a swine infection model [1,7-11]. The transcriptional response to *Salmonella *infection is predominantly a Th1 immune response as observed in porcine lung during *S*. Choleraesuis infection [9] and in porcine mesenteric lymph nodes (MLN) during *S*. Typhimurium and *S*. Choleraesuis infection [8,12]. The antigen processing- and apoptosis-related pathways were strongly induced at 24 and 48 hpi in porcine lung during *S*. Choleraesuis infection based on oligonucleotide microarray data [9]. Two important early proinflammatory cytokines, IL8 and IL1B, were rapidly induced in porcine jejunal and distal ileal Peyer's patches during *S*. Choleraesuis infection [10,11]. Uthe et al. (2005) used suppression subtractive hybridization (SSH) to identify genes which were up- or down-regulated at 24 hpi in porcine MLN during *S*. Choleraesuis infection compared to non-infected animals. Most of these genes have been annotated as being involved in host cellular functions including innate immunity and cytoskeleton regulation [7]. Skjolaas et al. (2006) tested the effect of *S*. Choleraesuis infection on gene expression in pig jejunal epithelial cells; IL8 and CCL20 were significantly induced at 1.5 h and 3 h post exposure, whereas CCL20 reached peak response at 6 h post exposure [13]. Recently, two publications described the use of an ileal loop model to explore the transcriptional response of pig intestinal mucosa to *S*. Typhimurium [14] and to both *S*. Typhimurium and *S*. Choleraesuis [15]. The former study showed very few gene expression differences in ileal tissue over 1–8 hpi, and IL8 was found to be induced only at 4 hpi [14]. However, the latter study showed that TNF*α*, IL8 and IL-18 was induced significantly during *S*. Typhimurium infection compared to serovar Choleraesuis at 24 hpi [15].

A comparison of the molecular pathways controlling host-pathogen interactions will not only increase our understanding of immune response biology, but may provide targets for drug development and genetic improvement of disease resistance. In this study, a genome-wide investigation of the porcine response to *S*. Choleraesuis infection was conducted using the Affymetrix GeneChip^® ^Porcine Genome Array containing oligonucleotides representing approximately 23,256 transcripts from 20,201 S. scrofa genes. We report the global transcriptional profile of the MLN, the largest lymph nodes in the human and animal body and one of the components of gut-associated lymphoid tissues (GALT), to *S*. Choleraesuis infection at the acute and chronic stages. We characterized the host immune responses with pathway analysis, targeting a better understanding of the host's innate immune pathways and regulatory targets in the MLN. We also confirmed the induction of many members of the NF*κ*B pathway during the infection and detected previously unidentified potential NF*κ*B target genes. We extended this broad screen for transcriptionally responsive genes by defining a set of gene as novel NF*κ*B targets genes as they are co-expressed with known NF*κ*B targets and whose orthologous human promoters contain NF*κ*B binding motifs.

## Results

### Clinical signs of disease

Clinical disease post-inoculation with S. Choleraesuis was observed in the animals in this study, and described previously [8]. Briefly, loss of appetite, lethargy and diarrhea of infected animals were observed at 48 hpi and continued until 9 dpi. The rectal temperature of infected animals peaked at 48 hpi (41.6 ± 0.4°C) and gradually declined to that seen in non-infected animals (around 39.7°C) by 8 dpi. Serovar Choleraesuis was detected in the ileocecal lymph nodes at 8 hpi (6.4 × 101 cfu/g), reached and maintained a level of 2–3 × 10^5 ^cfu/g from 24 h to 7 dpi., and declined to 2.1 × 10^3 ^cfu/g by 21 dpi.

### Transcriptome

The normal porcine MLN tissue transcriptome was represented by all probesets (14,348) which showed a Present call by the Affymetrix Microarray Analysis System 5.0 for all three non-infected animals. All probesets that showed a Present call for all three replicates in at least one time point during infection (15,935) were counted as the transcriptome of infected porcine MLN tissue. Combining these lists gave 16,046 probesets (70% of the whole chip) that detected expression in MLN [see Additional file 1].

### Differentially expressed gene analysis during *S*. Choleraesuis infection

Swine infected with *S*. Choleraesuis exhibit high bacterial loads as early as 24 hours post infection and reach maximal levels at 2–7 dpi [8]. Thus we have targeted our screen for responsive genes at the early time points of infection where unique control mechanisms might be revealed. Genes with a *p*-value < 0.01 and an estimated FC > 2 in at least one of the 10 time point pair-wise comparisons were declared to be differentially expressed. A total of 1,853 genes satisfied these criteria [see Additional file 2]. The largest *q*-value associated with this list of genes was 0.26, which is an estimated upper bound of the False Discovery Rate (FDR). Of these 1,853 differentially expressed genes, 1,189 genes (64%) have sequence similarity to a human Refseq based on our BLAST analysis using consensus sequences based on alignments of all publicly available porcine expressed sequence data (see Methods).

**Figure 1:**
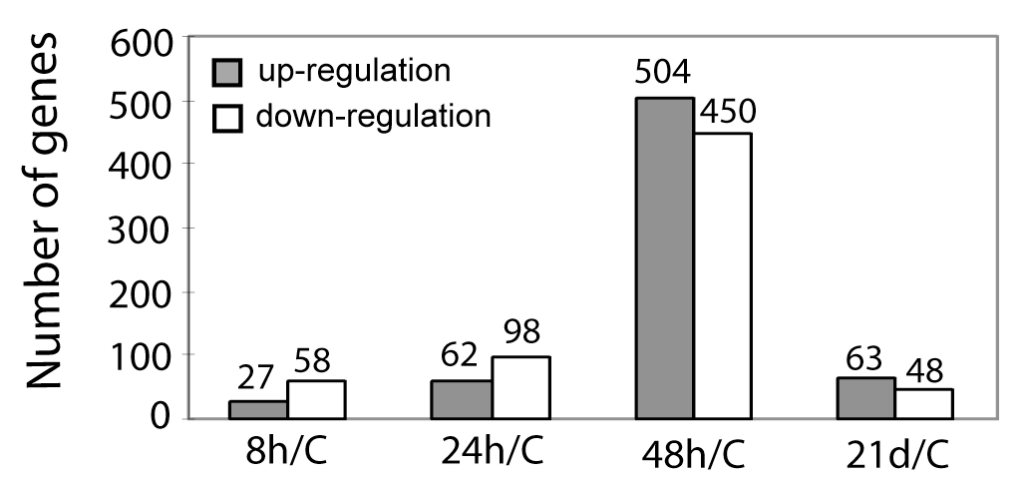
Differentially expressed genes at each time point during infection with *S*. Choleraesuis (*p *< 0.01, FC > 2, *q *< 0.26), compared to non-infected pigs.

Also, compared to non-infected pigs, the transcriptional response to *S*. Choleraesuis infection identified 85, 160, 954, and 111 transcripts differentially expressed (*p*-value < 0.01 and estimated FC > 2) at 8 hpi, 24 hpi, 48 hpi and 21 dpi, respectively (Fig 1). Clearly, a strong transcriptional response was observed at 48 hpi, with a similar number of up- and down-regulated genes. Table 1 shows 32 up-regulated and 9 down-regulated genes with a fold change greater than 10 at 48 hpi in infected MLNs, relative to non-infected MLNs (*p*-value < 0.01). Of the 30 annotated up-regulated genes in Table 1, 23 genes (77%) are involved in immune response, inflammation and apoptosis. The other 7 up-regulated genes, STEAP4, TNFAIP6, PSTPIP2, LIPG, HK3, STXBP1 and ITPR1, have not been previously associated with bacterial infection. The S100 calcium binding protein A9 (S100A9) exhibited the highest fold change (226.8) at 48 hpi, compared to non-infected pigs. A gene from the same family, S100A12, also showed strong up-regulation (38.9 estimated FC). Compared to the 32 up-regulated genes, only 9 transcripts were down-regulated using the same criteria of *p*-value < 0.01 and estimated FC > 10. Of these, only 3 genes (CA3, KRT17 and PLN) have been annotated, and none of these previously have been shown to be involved in the response to bacterial infection.

**Table 1:** Genes showing differential expression of large magnitude (*p*-value < 0.01 and estimated FC > 10) at 48 hpi as compared to uninfected pigs.

**affyID**	**Gene Name**	**Human Refseq ID**	***p *value**	**8 h/C**	**24 h/C**	**48 h/C**	**21 d/C**
Ssc.2381.1.A1_at	S100A9	NM_002965	0.0004	2.25	9.81	226.80	5.90
Ssc.719.1.S1_a_at	CXCL5	NM_002993	0.0015	3.09	6.28	102.29	4.41
Ssc.16008.1.S1_at	FCN2	NM_002003	0.0041	1.36	7.90	52.98	1.40
Ssc.17573.1.S1_at	IL1B	NM_000576	0.0000	1.16	3.70	39.31	1.15
Ssc.8162.1.S1_at	PTX3	NM_002852	0.0009	-1.40	2.61	39.10	1.05
Ssc.9117.1.S1_at	S100A12	NM_005621	0.0040	1.46	4.29	38.85	4.89
Ssc.16250.1.S2_at	IL1RN	NM_173841	0.0002	1.20	6.20	33.79	1.81
Ssc.658.1.S1_at	IL8	NM_000584	0.0002	2.13	3.17	31.80	2.33
Ssc.4093.1.A1_at	IFNG	NM_000619	0.0000	-1.27	1.26	28.83	-1.19
Ssc.13769.1.S1_at	LTF	NM_002343	0.0024	2.55	3.47	26.31	1.89
Ssc.9114.1.S1_at	STEAP4	NM_024636	0.0011	-1.25	2.92	20.85	2.83
Ssc.11009.1.A1_at			0.0033	-1.12	5.59	20.81	2.95
Ssc.4871.1.S1_at	CXCL2	NM_002089	0.0000	1.17	9.02	17.40	1.48
Ssc.300.1.S1_at	SLC11A1	NM_000578	0.0004	1.32	2.49	16.79	1.82
Ssc.16228.1.S1_at	PPBP	NM_002704	0.0003	-1.52	-1.38	16.17	-1.06
Ssc.5053.1.S1_at	CD163	NM_203416	0.0002	2.01	1.75	16.02	4.69
Ssc.27433.1.S1_at	TGM1	NM_000359	0.0003	1.97	3.38	14.78	1.56
Ssc.30887.1.S1_at	TNFAIP6	NM_007115	0.0009	-9.58	2.42	14.44	1.01
Ssc.30027.1.A1_at			0.0020	1.64	2.26	14.30	1.18
Ssc.18261.1.S1_at	PSTPIP2	NM_024430	0.0000	-1.02	1.68	13.30	2.20
Ssc.16151.1.S1_at	CSF3	NM_172219	0.0032	1.22	2.78	13.15	-1.15
Ssc.62.2.S1_a_at	IL6	NM_000600	0.0005	1.18	3.03	13.12	1.41
Ssc.21663.1.A1_at	LIPG	NM_006033	0.0001	1.35	2.94	12.95	1.78
Ssc.5743.1.S1_a_at	HK3	NM_002115	0.0002	1.04	1.94	11.64	1.32
Ssc.24282.1.S1_at	CXCL1	NM_001511	0.0000	-1.34	2.26	11.40	1.26
Ssc.6797.1.S1_at	STXBP1	NM_003165	0.0000	1.48	2.13	11.35	1.83
Ssc.11784.1.S1_at	TIMP1	NM_003254	0.0000	1.15	2.44	11.27	1.23
Ssc.7314.1.A1_at	PTGS2	NM_000963	0.0001	1.43	2.79	10.93	1.27
Ssc.30833.1.S1_at	CCL3	NM_021006	0.0033	2.36	3.01	10.67	1.88
Ssc.3706.1.S2_at	SOD2	NM_000636	0.0000	1.10	2.08	10.52	1.16
Ssc.7864.1.A1_at	IL1RAP	NM_002182	0.0000	-1.09	1.95	10.42	1.33
Ssc.16182.1.S1_at	ITPR1	NM_002222	0.0018	6.03	3.97	10.38	9.08

Ssc.18080.1.A1_at	C8orf46	NM_152765	0.0093	-1.45	-1.30	-10.34	-1.26
Ssc.10960.1.S1_at	CA3	NM_005181	0.0003	-1.17	-3.30	-10.50	-1.39
Ssc.8683.1.S1_at			0.0065	-1.11	-1.87	-10.61	1.26
Ssc.20230.1.S1_at			0.0036	-2.74	-1.53	-11.60	-1.37
Ssc.11065.1.A1_at	KRT17	NM_000422	0.0006	-2.06	-2.52	-11.93	1.15
Ssc.30225.1.A1_at			0.0007	-1.21	-2.54	-16.30	-1.13
Ssc.5227.1.S1_at	PLN	NM_002667	0.0034	1.06	-1.70	-18.57	1.80
Ssc.14164.1.A1_at			0.0000	-1.34	1.27	-22.76	1.32
Ssc.18150.1.A1_at			0.0027	-1.54	-4.65	-25.37	-1.25

### Cluster analysis

**Figure 2:**
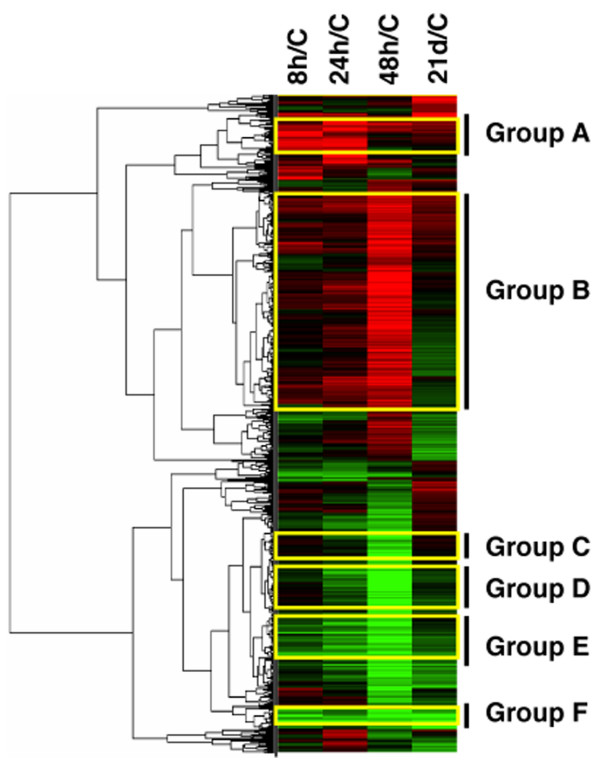
**Hierarchical clustering analysis of 1,853 genes from Affymetrix array analysis showing differential expression patterns during *S*. Choleraesuis infection.** Differential expression criteria: *p*-value < 0.01, estimated FC > 2, *q *< 0.26. The heat map was built by using Gene Cluster 3.0 software. Red color represents down-regulation and green shows up-regulation.

To get a broad overview of the changes in gene expression during infection and identify co-expressed gene clusters, a heat map was created using Gene cluster 3.0 software for the 1,853 differentially expressed genes. Two distinct clusters, an induced gene cluster and a repressed gene cluster, were identified and several interesting gene expression patterns were exhibited (Fig 2). Group A includes 52 genes that were repressed at 8 hpi and 24 hpi, after which RNA levels returned to those seen in the non-infected animals. Many ribosomal protein genes, as well as eukaryotic translation elongation factor 1 alpha 1(EEF1A1) were included in this group. A large number of genes that presented a comparatively lower expression level at 48 hpi during infection were found in group B. The annotated genes in this group had diverse biological functions, without an obvious overrepresentation of any specific pathway. A common feature of genes in group C, D and E is that they had a peak RNA response at 48 hpi during infection. However, genes in each group had different peak response times: 47 genes in group C, such as INFG, PSMB9, PSMB10 and C2, were only up-regulated at 48 hpi, while 141 genes in group D, such as TNF, IL6, IL8, IL1A and IL1B, increased their expression level at 24 hpi and expression peaked at 48 hpi. Genes in group E were slightly up-regulated as early as 8 hpi during infection; some heat shock proteins, such as HSPB1, HSPA6, HSPA1B were found in this group. An additional group F contained genes that were up-regulated at both the acute and chronic stages of infection, as compared to non-infected animals. This group included 38 annotated genes, including two complement related genes, C1QA and CFH.

### Gene Ontology annotation

**Figure 3:**
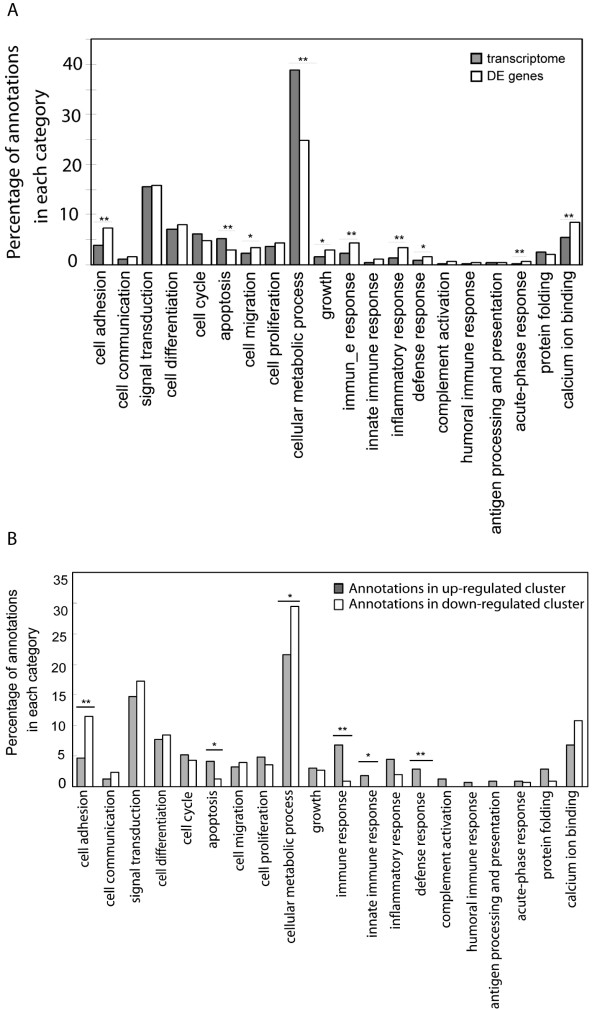
**Functional annotation of genes in the porcine MLN transcriptome and of genes differentially expressed (DE) between infected and non-infected pigs**. A. Gene Ontology (GO) annotation of the porcine MLN transcriptome and differentially expressed genes (*p *< 0.01, FC > 2, *q *< 0.26). All 16,046 probesets which were expressed in infected and non-infected porcine MLN (gray bars) and 1,853 differentially expressed genes (white bars) were annotated using our own specific GO-slim for biological processes and molecular functions relevant to immune response. The percentage of a given GO annotation across all annotations in the differentially expressed gene list, as compared to percentage annotation of the complete transcriptome, is shown. Statistical significance is denoted with an asterisk (*p < 0.05 and ** *p *< 0.01). B. GO annotations for up-regulated and down-regulated genes from induced and repressed gene clusters. Analysis details and statistics are denoted as in A.

Additional GO annotation of the 16,046 transcripts expressed in MLN were performed using our laboratory-designed GO-slim [12], built with terms in the biological process and molecular function categories of the GO database relevant for immune biology (Fig. 3A, gray bars). About 4,730 transcripts were assigned specific GO terms. Approximately 39% of these transcripts were annotated as being involved in cellular metabolic processes, and a significant number of transcripts were assigned known functions in signal transduction (15.6%), cell differentiation (7.2%), cell cycle (6.3%), calcium ion binding (5.6%), apoptosis (5.3%), cell adhesion (3.8%) and cell proliferation (3.7%). An additional 13% of the transcripts were assigned GO terms related to protein folding, immune response, cell migration, inflammatory response, defense response, antigen processing, and antigen presentation. The immune-oriented GO-slim annotation of the 1,853 differentially expressed genes was also performed (Figure 3A, white bars) and compared to the global transcriptome GO term assignment. The proportion of genes assigned GO terms associated with cell adhesion, apoptosis, immune response, inflammatory response, cellular metabolic process, calcium ion binding activity and acute-phase response was significantly enriched in our differentially expressed gene list (*p *< 0.01), compared to the transcriptome GO assignment.

An over-representation of a specific biological process does not indicate whether the process in question is being stimulated or repressed overall. To investigate over- or under-represented functional activities specifically within the up-regulated and down-regulated genes, GO annotations were also assigned to transcripts from the induced and repressed clusters again using the GO-SLIM [12] as above. Statistical analysis revealed that genes annotated with GO terms of immune response, innate immune response, defense response, apoptosis and cellular metabolic process were significantly enriched in the induced cluster, while the repressed cluster had a significantly higher percentage of genes related to cell adhesion (*p *< 0.05) (Figure 3B).

### Pathway analysis

**Figure 4:**
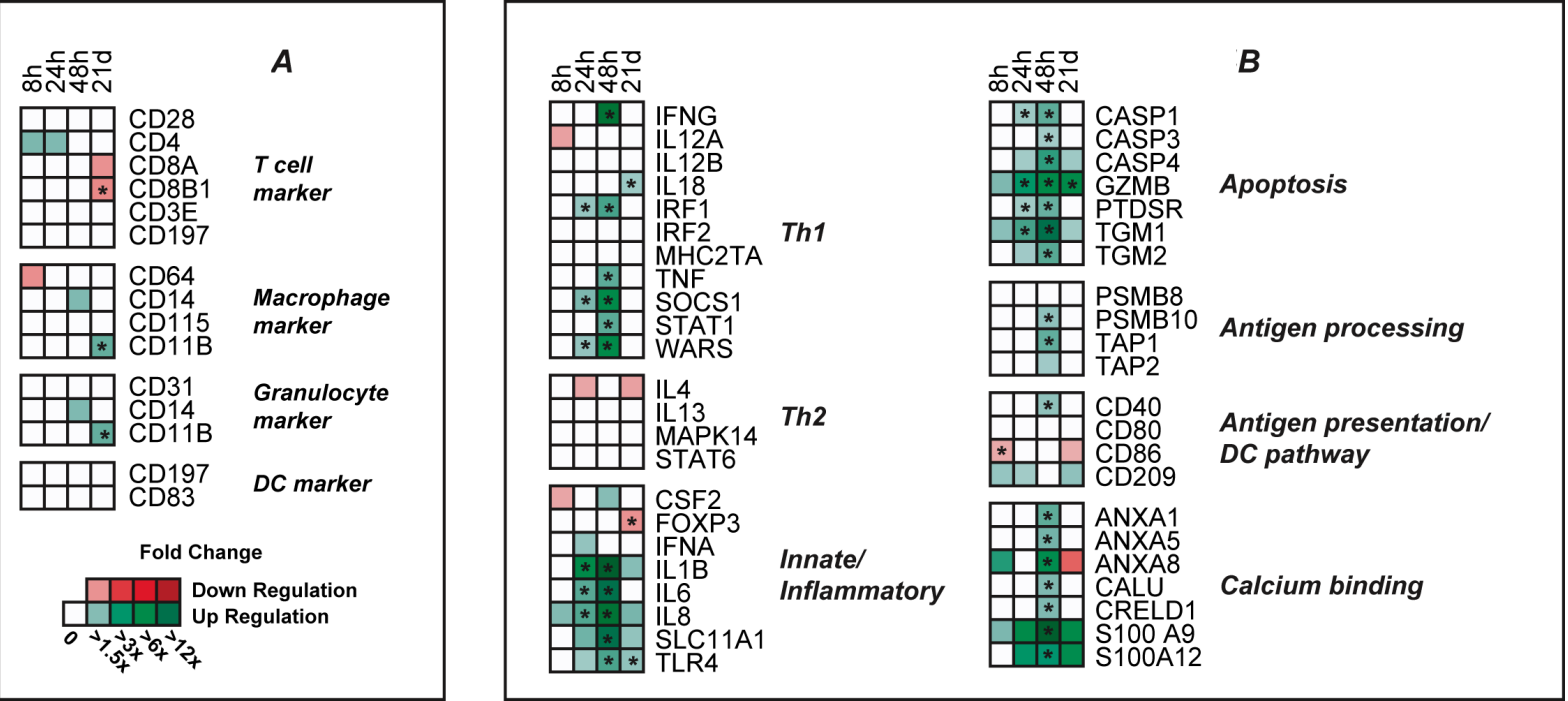
**Transcriptional profile of selected cell-type marker and immune response pathway genes by Affymetrix DNA microarray analysis.** Expression patterns of specific marker genes for T cell, macrophage, granulocyte and dendritic cell types (A) or important immune response pathways (B) are shown. The fold change from comparisons of infected pigs and non-infected controls at each time point were calculated from the Affymetrix array data using Genecluster. Statistical differences (*p *< 0.05) between control and infected pigs are represented by an asterisk (*).

During bacterial infection, T cells will migrate into the T-cell zone of the MLN and scan the surface of the antigen-presenting cells, primarily macrophages and dendritic cells, for specific peptide:MHC complexes [16]. The levels of any RNA transcript present in the MLN can be changed by cells migrating into or out of the lymph node, and these RNA changes might be erroneously interpreted as transcriptional response to infection within an immune cell type. To verify that the observed differences in gene expression are true transcriptional differences, Affymetrix data-based expression levels of specific markers for T cells, macrophages, dendritic cells and granulocytes were checked. No evidence of major changes in cell migration was observed since most RNA levels for these marker genes did not change significantly (Fig 4A). As we have further concentrated our analyses and interpretations on genes with large differences in expression, these data indicate the RNA differences we observed are likely representative of specific transcriptional responses within cells, and not due to significant changes in the abundance of specific cell types in the MLN.

To investigate immune-related pathways stimulated during infection, the Affymetrix-based expression patterns of genes that are known to be involved in specific immune pathways were collected (Fig 4B). Results showed that 6 out of 11 selected Th1-related genes, such as INFG, IRF1, SOCS1, STAT1, TNF and WARS, were significantly up-regulated at 48 hpi, while genes known to be predominately associated with Th2 response (IL4, IL13, MAPK14 and STAT6), were down-regulated or unchanged respectively at all time points during infection (Fig 4B). These data suggest that *S*. Choleraesuis elicited primarily a Th1-associated response within the MLN during infection.

Most genes known to be involved in innate/inflammatory pathways in Fig 4B, such as IL-8, IL6, SLC11A1, TLR4 and IL1B, increased their RNA expression level significantly at 24 hpi and/or 48 hpi during infection. Strong induction of apoptosis pathways was also observed in response to *S*. Choleraesuis infection. Seven apoptosis related genes (CASP1, CASP3, CASP4, GZMB, PTDSR, TGM1 and TGM2) were up-regulated significantly at 24 hpi and/or 48 hpi.

Two antigen-processing related genes, PSMB10 and TAP1, were induced at 48 hpi, which indicates that the antigen processing pathway was activated. However, *S*. Choleraesuis infection did not appear to significantly influence the antigen presentation/DC function pathway, because two genes known to be involved in dendritic cell activation, CD80 and CD86, did not show elevated expression level at either 24 or 48 hpi. In addition, CD209, which is also named DC-SIGN, showed non-significant changes during infection (*p *> 0.05).

**Figure 5:**
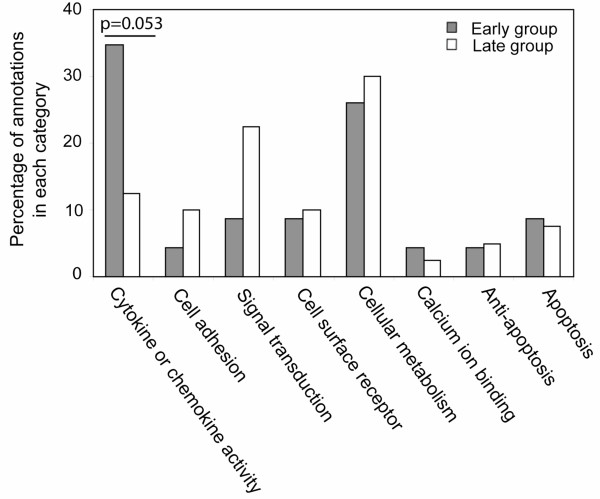
**Gene Ontology analysis of Early and Late Groups of Genes Responding to Salmonella**. The GO-slim described in Figure 3 and Methods was used for this analysis. The percentage of a given GO annotation across all annotations in the Early gene list, as compared to percentage annotation of the Late gene list, is shown.

We observed a significant induction of genes at 48 hpi that have GO annotation of calcium binding activity, including S100A9, S100A12, several ANXA family genes, and CALU. Therefore, we speculate that calcium pathways were strongly affected by infection at this time (Fig. 4B). Further, annotation showed that many of the genes that were up-regulated at 24 and 48 hpi are known direct NF*κ*B targets ([17]; ; ). These 58 known NF*κ*B genes are listed in Additional file 3. To determine whether NF*κ*B dependent genes formed a central part of the host transcriptional response to *S*. Choleraesuis infection, GO analysis was performed to compare the functional activities of these differentially expressed known NF*κ*B target genes from an "Early" group, defined as genes that were up-regulated significantly at 8 or 24 hpi (84 genes; *p*-value < 0.01 and estimated FC > 2), and a "Late" group, defined as genes that were only up-regulated at 48 hpi (324 genes; *p*-value < 0.01 and estimated FC > 2). Figure 5 demonstrates that cytokine or chemokine activity GO terms found in the known NF*κ*B target genes (such as CXCL1, CXCL2, CXCL6, IL1A, IL1B, IL1RN and CSF3), were enriched in the Early group as compared to the Late group (Fisher's exact test *p *= 0.053). The Late group NF*κ*B target genes were predominantly annotated in the signal transduction and cell metabolism categories.

### Q-PCR analysis of differentially expressed porcine genes

**Figure 6:**
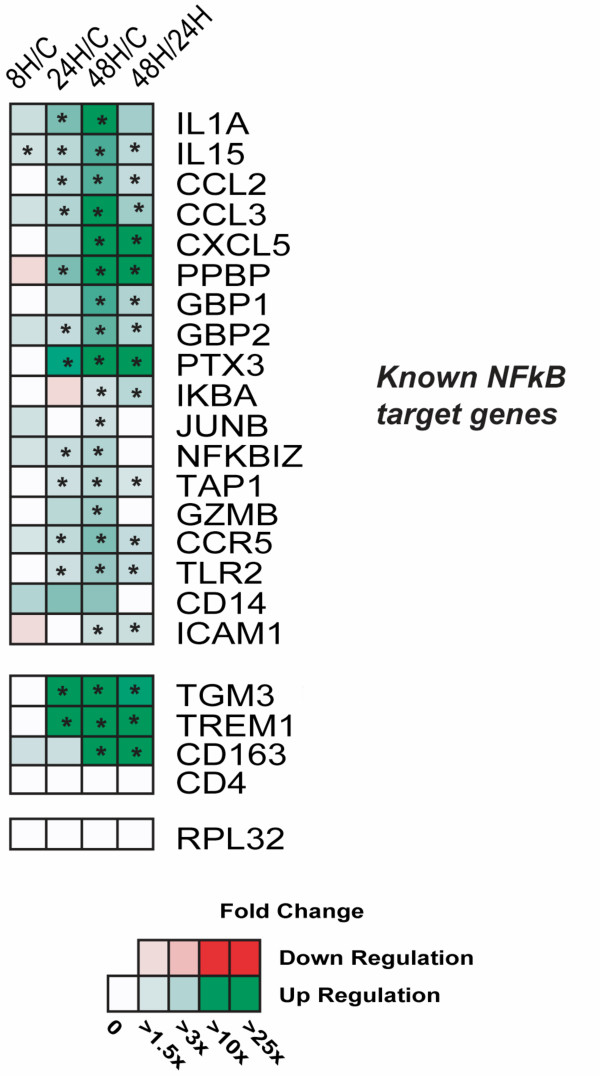
**Quantitative PCR analysis validates transcriptional profiling data for genes responding to *S*. Choleraesuis infection.** Real-time Q-PCR data is presented as the fold change in gene expression in infected pigs as compared to the negative controls. In the last column, gene expression from 48 hpi is compared to that at 24 hpi. Statistical significance (*p *< 0.05) is denoted with an asterisk (*).

To confirm the NF*κ*B genes that were declared differentially expressed in our microarray analysis, a panel of 22 genes was selected for real-time PCR analysis and validation of the expression patterns at 8 hpi, 24 hpi and 48 hpi. The RNA levels of 18 known NF*κ*B target genes (IL1A, IL15, CCL2, CCL3, CXCL5, PPBP, GBP1, GBP2, PTX3, IKBA, JUNB, NFKBIZ, CD14, ICAM1, TLR2, GZMB, TAP1 and CCR5) were measured. We also analyzed the expression for a T cell marker gene (CD4) and a macrophage marker gene CD163. Although an oligonucleotide set representing the TGM3 gene could not be found on the Affymetrix microarray, Q-PCR was performed for this gene since our earlier work [9] showed a strong up-regulation of TGM3 in porcine lung during acute infection with *S*. Choleraesuis, and the temporal expression pattern is similar to known NF*κ*B target genes. Finally, TREM1 was also selected for Q-PCR validation because our microarray data showed its expression pattern to be similar to known NF*κ*B target genes, although no reports have shown that TREM1 is directly regulated by NF*κ*B. Comparison of the QPCR results with the microarray data demonstrated that expression differences for 20 of the 21 genes were statistically significant by QPCR, confirming the Affymetrix-based results for these genes and RNA samples (Table 2 and Fig. 6). Results showed that, except for CD14, all known NF*κ*B-regulated genes were significantly up-regulated at 48 hpi during infection, and many of the genes experienced a strong induction from 24 hpi to 48 hpi. We also confirmed that TGM3, TREM1 and CD163 exhibited a similar transcriptional profile with many known NF*κ*B target genes.

**Table 2:** Q-PCR results for gene expression (C_t _values, SD) at each early response stage (8 hpi, 24 hpi and 48 hpi) in *S*. Choleraesuis infection.

**Gene name**	**SC – Control**			**SC – 8 h**			**SC – 24 h**			**SC – 48 h**		
	
	**Average**	**SD**	**Stat**	**Average**	**SD**	**Stat**	**Average**	**SD**	**Stat**	**Average**	**SD**	**Stat**
IL15	26.5	0.67	A	25.7	0.38	B	25.0	0.55	B	23.7	0.14	C
CCL2	21.6	1.27	A	21.4	0.34	AB	20.0	0.47	C	18.8	0.24	D
CCL3	29.0	1.03	A	28.2	0.09	AB	27.4	0.27	B	25.5	0.97	C
CCR5	27.5	1.12	A	26.9	0.36	AB	26.2	0.67	B	25.1	0.31	C
GBP1	22.8	0.97	A	22.2	0.33	A	21.6	0.98	A	19.9	0.49	B
GBP2	24.4	0.68	A	23.7	0.23	AB	23.3	0.60	B	21.8	0.22	C
ICAM1	29.0	0.60	A	29.6	0.35	A	29.1	0.19	A	28.0	0.18	B
TAP1	22.2	0.57	A	22.4	0.35	A	21.3	0.17	B	20.7	0.27	C
TLR2	25.6	0.83	A	25.4	0.04	AB	24.7	0.52	B	23.5	0.21	C
TGM3	31.8	0.67	A	31.3	0.48	A	25.7	2.64	B	22.6	0.11	C
GZMB	23.5	0.92	A	23.3	0.62	AB	22.1	0.64	AB	21.6	0.39	B
CD14	28.3	0.82	A	26.8	2.77	A	26.0	4.17	A	26.2	0.54	A
IL1A	28.9	0.85	A	27.9	0.56	AB	26.5	1.61	BC	24.7	0.31	C
PPBP	34.3	1.10	A	35.0	0.71	A	31.9	2.13	B	23.9	0.53	C
JUNB	30.8	0.68	A	30.1	0.17	AB	30.3	0.79	AB	30.0	0.26	B
IKBA	25.6	0.63	A	25.6	0.39	A	26.3	0.54	A	24.8	0.31	B
NFKBIZ	23.4	0.69	A	22.7	0.68	AB	22.3	0.40	B	21.8	0.37	B
CD163	26.5	0.90	A	25.7	0.49	A	25.5	1.15	A	21.4	0.84	B
CXCL5	29.4	0.96	A	28.8	0.21	A	27.8	2.06	A	23.2	0.53	B
PTX3	28.7	0.71	A	28.6	0.72	A	25.7	2.54	B	22.0	0.25	C
TREM1	30.5	0.91	A	30.5	1.21	A	27.1	1.97	B	23.6	0.44	C
CD4	23.8	0.68	A	23.3	0.10	A	23.3	0.64	A	23.4	0.62	A
RPL32	16.9	0.34	A	16.9	0.14	A	16.8	0.31	A	17.1	0.37	A

Expression values not sharing the same letters are statistically different across time points.

### Identification of putative NF*κ*B target genes within the differentially expressed gene lists through bioinformatic analysis of the human orthologous promoter sequences

To further explore the possibility that the response observed might have identified novel NF-*κ*B target genes, we collected *in silico *comparative evidence that NF*κ*B may bind to flanking sequences of these genes [see Additional file [Supplementary-material S4]]. We used human genomic sequence data for the differentially expressed genes in the Early group (83 human orthologs found for 84 Early genes; E83), the Late group (319 orthologs found for 324 Late genes; L319), and all up-regulated genes to identify those genes containing statistically significant NF*κ*B binding sequence motifs near their promoters. The latter group contained 560 genes total, with 544 orthologs found, of which we used only 500 human orthologs (A500) in the TFM-Explorer analyses [18], as this was the maximum allowed by TFM-Explorer. However, all 544 were used in Clover [19] as a single group; A544. We also investigated the effect on finding putative NF*κ*B targets in these groups when removing the known NF*κ*B target genes from these groups (Methods; Additional file 3), to explore the underlying regulatory "signal" in these promoters. Thus we generated an early group without 22 known targets (E61), a Late group with 36 known NF*κ*B targets removed (L283), and a complete group of all up-regulated differentially expressed genes with the 69 known NF*κ*B targets removed (U475).

**Figure 7:**
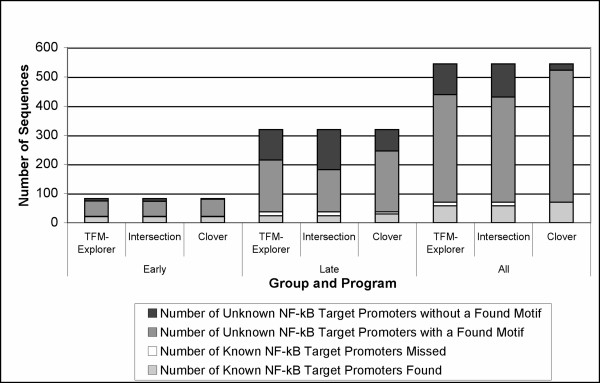
**Computational identification of NF*κ*B binding site motifs at human orthologs of genes from Early, Late, and a combined All groups predict putative NF*κ*B genes responding to *S*. Choleraesuis.** Each bar graph shows (for Clover, TFM-Explorer, or the intersection of these results) for each group: a) the number of known target genes whose promoter contains a NF*κ*B motif (light gray), b) the number of known target genes whose promoter was not identified as having a NF*κ*B motif (white), c) the number of unknown NF*κ*B target genes whose promoter was identified as having a motif (dark gray), and d) the number of unknown target genes whose promoter was not identified as having a NF*κ*B motif (black).

TFM-Explorer analysis of E61, representing all genes up-regulated in the first 8–24 hpi but with known NF*κ*B targets removed, found 51 promoters (84%) with at least one NF*κ*B binding site (data not shown). However, when the full E83 set, including all known NF*κ*B targets, was analyzed, 73 sequences (88%) were identified with one or more NF*κ*B binding sites (Fig 7). The software identified almost all of the known targets (21 of 22). Similarly, TFM-Explorer analysis of L283 (Late genes with known NF*κ*B target genes removed) identified 178 sequences with target sites, while analysis of L319 identified 202 sequences with NF*κ*B sites, including 24 out of 37 known NF*κ*B targets. Similar analysis of U475 identified 300 gene promoters with NF*κ*B motifs, while an average of the results for testing five A500 groups (iterated sampling of the complete 560 up-regulated and annotated complete gene set) shows 310 sequences were identified (totaling 427 putative targets), which on average found 45 of the 70 known NF*κ*B target genes in this group (totaling 58 when summed across all five replicates) (Fig 7).

Clover analysis shows similar results using 5,000 permutations and *p *< 0.01 as a significance level cut-off. In the E83 group 80 of the 83 input sequences were found to have a NF*κ*B binding sequence, with 21 of the 22 known targets being found. For L319, Clover identified 239 putative targets including 30 out of the 37 known targets. When all differentially expressed genes are grouped together, (A544), 528 promoter regions were identified to contain a putative binding site; finding 70 out of 70 known targets. Taking the intersection of TFM-Explorer and Clover results, there are 72 promoter sequences with a potential NF*κ*B binding site in the Early group. Within these 72 promoters, 21 are known to contain a binding site, leaving 51 potential novel NF*κ*B targets. Similarly, for the Late group 169 promoters were found by both programs, and as 24 of the promoters are known NF*κ*B targets, this identifies 145 potential new direct targets of NF*κ*B. Combining the results for only the Early and Late groups, 241 total promoters were predicted to have NF*κ*B binding sites. Across the complete set of differentially expressed genes, 419 promoter sequences were found by both programs, with 58 being previously known targets of NF*κ*B, yielding 358 potential new NF*κ*B targets (Fig 7). All genes in the Early, Late and All categories, as well as whether or not a motif was found for each gene, are shown in Additional file 4.

## Discussion

### Transcriptome, differentially expressed genes and GO annotation

In this study, the Affymetrix GeneChip^® ^Porcine Genome Array and Q-PCR were used to monitor the whole-genome expression profile of porcine MLN in response to *S*. Choleraesuis infection. Quite similar to the porcine MLN transcriptome seen in a study on the response to *S*. Typhimurium [12], expression of more than 16,000 transcripts were reproducibly detected and of these, 11,020 (68.7%) transcripts had a significant hit to human RefSeq. Thus, we believe the transcriptional response detected in our study represents a high proportion of the porcine genomic response to *S*. Choleraesuis infection within the MLN. A technical limitation of this work is that the Affymetrix chip cannot assay for transcript that are not represented by probesets. An analytical limitation is that we primarily analyzed only those genes with a relatively large fold change, due to the large number of genes showing differential expression. While a significant number of genes passed our filters, there are additional genes and possibly pathways yet to be analyzed in these data, and all data is provided in Additional files and to NCBI-GEO. Statistical analysis of differential expression revealed 1,853 genes changed their expression level within at least one of 10 possible pair wise comparisons during infection, and about 63% of these differentially expressed genes were annotated using BLAST analysis. Limitation of availability of full-length porcine cDNA and the fact that many human/mouse genes do not have functional annotation, the non-annotated genes are currently not very helpful in furthering our understanding of gene functions and pathways responding to infection. However, the discovery of these non-annotated genes as part of the transcriptional response to bacterial infection is novel, and, as better annotation becomes available, will contribute to an improved understanding of the pathways of host response to bacteria infection across host species.

Compared to the response in porcine MLN during the *S*. Typhimurium infection, where only 97 genes were induced significantly at 48 hpi [12], a strong host transcriptional response at 48 hpi with *S*. Choleraesuis was observed, as 954 genes were differentially expressed at 48 hpi compared to non-infected pigs (Fig 1). This is consistent with the clinical manifestations that the rectal temperature of infected animals peaked at 48 hpi. As expected, we found many genes with large gene expression changes, such as cytokines, chemokines and heat shock proteins; these generally overlap with sets of genes that have been implicated in host response to infection by others [20,21], indicating our data can be integrated with similar results in other species. However, we also identified a number of genes that had not previously been shown to be involved in host response to bacterial infection, such as STEAP4, PSTPIP2, LIPG, HK3, STXBP1, ITPR1, CA3, KRT17 and PLN. None of these genes responded significantly to *S*. Typhimurium during infection [12]. Thus we predict that these induced genes are specific to the response to the *S*. Choleraesuis serovar, as compared to *S*. Typhimurium. Even though the biological function of these genes during infection remains unclear, these data more fully describe the transcriptional response to *S*. Choleraesuis and suggest additional functional roles for these genes. Furthermore, the differentially expressed genes with known immune functions and those with unknown functions add to the list of candidate genes to investigate for associations between immune related traits and DNA-level variation; polymorphisms at these candidate genes might result in valuable markers for enhancing disease resistance, pig health, and food safety through molecular breeding methods. Even though we are focusing on the early response (8, 24 and 48 hpi) during the infection, the genes expressed differentially at day 21, include CCL5, CCR5, TGFB3 and AIF1, and provide additional gene profiling data during the chronic infection stage.

### Cluster analysis

Co-expression of genes of known function with poorly characterized or novel genes may provide a simple means to gain leads as to the functions of genes for which information is not currently available. In our study, hierarchical cluster analysis was performed on 1,853 genes that were differentially expressed during infection, and we find the heat map was driven by the 954 differentially expressed genes at 48 hpi. A similar heat map analysis of the transcriptional response to *S*. Typhimurium shows that the major peak of 151 responding genes occurred at 24 hpi during infection [12], thus we propose that porcine MLN had a different response peak time for *S*. Typhimurium and *S*. Choleraesuis infection. This is consistent with the results from a previous study using SSH technology to investigate expression at 24 hpi [8]. As we observed in porcine MLN response to *S*. Typhimurium [12], some ribosome- and/or translation-related genes were repressed at 8 hpi and 24 hpi. This effect is also similar to the response to LPS in skeletal muscle of neonatal and adult pigs [22,23] and to the response to endotoxin in human blood leukocytes [24], where a large number of genes involved in translation were repressed. Thus, we speculate that an early pathogenic effect of multiple serovars of *Salmonella *on the host is suppression of translation.

Three gene groups (Fig. 2, group C, D and E) in the induced cluster exhibited different expression patterns but shared a common feature, a peak expression response at 48 hpi. As large numbers of NF*κ*B target genes were found in groups C and D, we discuss their expression features in the context of NF*κ*B signaling pathways below.

### Pathway analysis

A predominant Th1 immune response was observed during infection, as 6 of 11 investigated Th1 associated genes, including TNF, IFNG, and some IFNG-signaling responsive genes (SOCS1, STAT1, WARS and IRF1), were strongly up-regulated at 24 hpi and/or 48 hpi (Fig. 4B). This is consistent with the results in porcine lung during *S*. Choleraesuis infection [9] and in porcine MLN in both *S*. Typhimurium and *S*. Choleraesuis infection [8,12]. However, IL12A and IL12B, which are thought to also favor Th1 cell development, did not change their expression level during infection. Their low expression level in response to *S*. Choleraesuis has also been detected by QPCR analysis of infected porcine MLN by our group [8]. A low expression level of IL12 has been also observed in the pig in response to porcine reproductive and respiratory syndrome virus [25] and in pig MLN response to *S*. Typhimurium infection [12] and *Toxoplasma gondii *[26] infection. We speculate that lack of IL12 induction during *S*. Choleraesuis infection might stifle IFNG induction and negatively affect host defense against *Salmonella*.

Our microarray data shows that apoptosis pathway related genes displayed a strong induction at 24 hpi and reached a peak response at 48 hpi in response to *S*. Choleraesuis infection. Multiple genes within gene families involved in apoptosis pathways were induced significantly, such as genes in the caspase family (CASP1, CASP3 and CASP4) and in the transglutaminase family (TGM1, TGM2 and TGM3). The caspases are a family of cysteine proteases important not only in starting and executing apoptosis, but also in processing and maturation of the inflammatory cytokines IL-1b and IL-18 [27]. The CASP1 gene is known to be important in inflammation due to its role in maturation of IL-1b and IL-18. Even though it is unclear whether CASP1 plays a direct role in apoptosis, overexpression of CASP1 has been shown to cause apoptosis in a variety of cell lines [28]. A recent study revealed that IPAF is an activator of CASP1 and IL-1b in *Salmonella *infected macrophages [29]; unfortunately, we did not find any oligonucleotide set representing the IPAF gene on the Affymetrix microarray. Like CASP1, CASP4 has been shown to process pro-IL-18 and IL-1F7b, albeit inefficiently, and to cleave CASP3 into its active form. One important characteristic of CASP4 is its robust induction by INFG and the NF*κ*B complex [30,31].

Genes from the transglutaminase family can be significantly induced in porcine lung during *S*. Choleraesuis infection [9] and in porcine MLN during *S*. Typhimurium infection [12]. In this study, TGM1, TGM2 and TGM3 showed exactly the same expression pattern: activation was initiated at 24 hpi and peaked at 48 h post *S*. Choleraesuis infection in porcine MLN. The TGM3 gene showed a very strong induction at 48 hpi, with the fold change about 600 compared to non-infected animals by Q-PCR. There is evidence that TGM2 is a NF*κ*B dependent gene [32], and researchers have shown that increased TGM2 activity can trigger NF*κ*B activation through an unusual I*κ*B polymerization reaction rather than I*κ*B degradation through IKK signaling [33]. Although transglutaminase genes have been demonstrated to elevate their expression level during inflammation and to play a physiological role in mediating defense against injury or infection in various cell types [33], their role in apoptosis is not yet clear.

Another important cellular immune response to infection that occurs in the MLN is antigen processing and presentation, as phagocytic cells in the MLN communicate with T cells for further immune activation and initiation of adaptive immune responses. Three of four antigen processing related genes (Fig. 4B) exhibited an increased expression level at 48 hpi, which is consistent with the gene expression patterns that were observed in porcine lung during *S*. Choleraesuis infection by [9]. Interestingly, two markers involved in the antigen presentation activation pathway, CD80 and CD86, did not show significant expression changes at 24 hpi and 48 hpi. These data tempt us to speculate that the host DC-mediated antigen presentation pathway was altered early after infection. Antigen presentation by murine DC cells can be inhibited by *S*. Typhimurium [34,35], and we observed that both CD80 and CD86 were down-regulated at 8 hpi in *S*. Typhimurium infected MLN [12]. Thus, we predict that lack of a strong DC-mediated antigen presentation might be a mechanism that permits *S*. Choleraesuis to escape the porcine GALT and cause a systemic infection.

One characteristic of host MLN transcriptional response to *S*. Choleraesuis is that several groups of genes with annotations for calcium binding activities changed their RNA expression levels significantly during infection. Calcium binding proteins are important molecules in the transduction of calcium signaling, which evoke various cellular processes, such as cell migration, cell differentiation, cell death and cell growth [36]. The S100 gene family is the largest group of calcium binding proteins; two members of this gene family, S100A9 and S100A12, were up-regulated significantly with a large fold change at 48 hpi relative to non-infected animals in our study. Induction of S100A9 has been also observed in porcine Peyer's patch in response to *S*. Choleraesuis infection [11]. Overexpression of S100A9 and S100A12 at the site of inflammation has been well described in humans, and S100A8/S100A9 and S100A12 are used as clinical laboratory markers for inflammation [36]. The exact biological function of these genes remains to be defined in greater detail, although there is evidence that they have anti-microbial properties [37], and are involved in induction of apoptosis [38]. Other genes with GO annotation for calcium binding activity, including annexins (ANXA1, ANXA5 and ANXA8) and transglutaminases (TGM1, TGM2 and TGM3), showed differential expression at 24 and/or 48 hpi compared to non-infected animals. Calcium is an important second messenger in cells and changes in calcium pathways can evoke various cellular processes [36]. Our report is the first to describe the strong induction of multiple calcium binding protein genes in MLN of *S*. Choleraesuis infected pigs and adds new information to host transcriptional response to gram-negative bacteria infection in pig and other species.

### NF*κ*B signaling pathway

NF*κ*B is a latent transcription factor held in the cytoplasm through binding by its inhibitors I*κ*B. During stimulation or infection, I*κ*B is phosphorylated and degraded, allowing NF*κ*B translocating into the nucleus to induce expression of many genes [17]. The NF*κ*B target genes control a variety of cellular processes. Both microarray and QPCR data analysis revealed that many known NF*κ*B target genes were significantly up-regulated from 24 hpi to 48 hpi, indicating a strong NF*κ*B response during acute *S*. Choleraesuis infection. Both the magnitude and timing of this response was different from the response to *S*. Typhimurium, where suppression of the NF*κ*B pathway from 24 to 48 hpi was observed [12]. Thus understanding the transcriptional profiles of NF*κ*B target genes is an important step to further understand its regulatory function in bacterial infection. Different activation times for NF*κ*B target genes during stimulation has been intensively studied [39,40]. Analysis of LPS-stimulated mouse macrophages demonstrated that NF*κ*B binding occurs in two distinct waves due to different rates of NF*κ*B recruitment [39]. Tian et al. (2005) identified "Early", "Middle" and "Late" expression profiles of NF*κ*B target genes during the TNF stimulation in epithelial cells, since these genes have peak response at 1 h, 3 h and 6 h, respectively. Three mechanisms for these differential expression patterns of NF*κ*B target genes were suggested; a) Early and Late gene promoters are bound by NF*κ*B complexes containing different subunits; b) there are different environments in which the NF*κ*B binding sites are located between the Early and Late gene promoters or c) genes in the Late group undergo an additional rate-limiting step necessary for promoter activation [40].

In our study, two groups of NF*κ*B target genes were identified, an ''Early'' group and a ''Late'' group. To determine the biological functions of genes in the Early versus the Late groups, GO annotations were assigned. GO terms for cytokine activity or chemokine activity were enriched in the Early genes (Fisher's exact test *p *= 0.053), while the Late group was predominantly composed of genes with signal transduction and cell metabolism annotations. This is consistent with the functional categorization of NF*κ*B dependent genes in ''early'' and ''late'' groups of [40]. The rapid induction of cytokines and chemokines during infection clearly may be important for recruiting immune cells to infection sites.

Both microarray and Q-PCR data suggested that NFKBIA (which encodes I*κ*B*α*) increased its RNA level slightly at 48 hpi. There is overwhelming evidence that demonstrates that expression of this inhibitory gene is activated by NF*κ*B in a negative feedback loop, which provides an effective mechanism for controlling NF*κ*B activity [41]. It has also been shown that NF*κ*B DNA binding activity is at its peak when NFKBIA is newly synthesized [39]. Thus, as we observe up-regulation of NFKBIA and strong activation of other NF*κ*B target genes at 24 hpi, we speculate that NF*κ*B activity was initiated at 24 hpi and transcriptional responses to NF*κ*B peaked at 48 hpi. This contrasts to the response of NF*κ*B target genes to *S*. Typhimurium infection, where NFKBIA was not up-regulated significantly at 48 hpi, and many genes were suppressed from 24 hpi to 48 hpi [12].

### Identification of potential porcine NF*κ*B targets

Using TFM-Explorer and Clover, it was possible to identify promoter regions containing NF*κ*B motifs in human genes orthologous to the porcine genes which had Early (within 24 hpi) and Late (by 48 hpi) response to *Salmonella *infection. We have confidence that our analysis has identified strong candidate NF*κ*B target genes, as the cutoff for identifying NF*κ*B motifs within promoters was stringent, given that the non-zero possibility that groups of randomly selected genes used as the background in the TFM Explorer analysis will contain some NF*κ*B targets. Some of the known targets were missed by our TFM-Explorer analysis, but the input sequence window used was only 2,000 total bases. Thus for these known targets, there might be some binding sites outside of the input sequence range. However, we were able to identify 95%, 65% and 83% of the known targets in the E83, L319 and A544 groups, respectively, indicating that our sensitivity was adequate to find the large majority of known target genes.

Results for DNA regulatory motif analysis of human orthologs for the stimulated gene groups provided evidence for 51 putative NF*κ*B targets in the Early group and 145 in the Late group. These putative targets were also analyzed by GO annotation to further develop our understanding of the regulatory response to *Salmonella *infection (data not shown), as performed on the known NF*κ*B target genes (Figure 5), These putative target genes, including SOCS1, SCARB2, CEBPD, CXCL16, IL1RAP, and CASP7, are involved in multiple cellular processes, including cell adhesion, regulation of transcription, immune response, and receptor activity. While there is no additional experimental evidence that these genes are direct NF*κ*B targets, many of them have been previously reported to be involved in the response to bacterial infection [8,42,43]. Further, the motif-finding software TFM-Explorer was able to predict nearly all of these promoters as containing NF*κ*B motifs, even when known NF*κ*B target promoters were removed from the test set. This indicates that these putative NF*κ*B target genes have important regulatory regions in common with bona fide NF*κ*B targets, and that these common motifs can be found independently in the known motif data. Thus, although further experimental work is needed to confirm that these genes are true NF*κ*B targets, our analysis detecting NF*κ*B regulatory motifs in these genes further supports the hypothesis that they constitute an important part of the early anti-bacterial infection response.

To supplement our PubMed literature search that found none of these genes were known NF*κ*B direct targets, Pathway Studio (a PubMed text-mining software) analysis was run on the complete sets of Early and Late genes. Pathway Studio identified seven Early group genes having literature evidence of direct binding of NF*κ*B to their promoter region (CEBPD, IL1B, IL6, IL8, FOS, EGR1, PTGS2), while in the late group only two were identified as having literature evidence of direct binding (TNF, IFNG). In addition, in the Early group, ten other genes were identified as having a connection with NF*κ*B in the literature, usually sharing a common expression pattern (CSF3, CYCS, OAS1, CEBPB, CELP, IER3, SOD2, CCL2, HLA-A, CXCL2). For the Late group, an additional 11 were also found to have a connection with NF*κ*B (LYZ, SP6, IL10, TLR2, BCL2, APP, HXB, CCR5, ANXA5, IL15, CDKN1A). TFM-Explorer and Clover found motifs at the promoters of: a) all genes with direct binding evidence; b) all but one of the NF*κ*B-connected genes (CYCS) identified in the Early group (94%); and c) all but two (CCR5, ANXA5) of the 13 (77%) in the Late group. Of the genes that were predicted to contain an NF*κ*B binding site by both programs and had evidence from Pathway Studio as having a direct binding relationship with NF*κ*B, only CEBPD and EGR1 were not previously on the "known" list of targets at available websites (see Methods). However, due to more recent literature evidence provided by the Pathway Studio software, they should be considered "known" NF*κ*B targets. Thus, this literature analysis indicates that the large majority of genes predicted to be NF*κ*B targets (Fig 7), based on co-expression and motif data, have not yet been recognized as direct NF*κ*B target genes. As this evidence is based on human promoter sequences, these genes may be regulated by NF*κ*B during cellular responses to *Salmonella *in other species as well, including human.

## Conclusion

This study investigated the host transcriptional response to *S*. Choleraesuis infection in porcine MLN using the Affymetrix porcine GeneChip^® ^Transcriptome. A large number of differentially expressed genes (*p *< 0.01, FC > 2) were identified and functional analyses of these genes was performed by GO annotation enrichment calculations. Gene expression hierarchical cluster analysis and specific pathway analysis revealed several specific features of porcine host response to infection, and expanded the gene targets for future genomic studies. Comparison of the magnitude and timing of porcine MLN transcriptional response to different *Salmonella *serovars, *S*. Choleraesuis and *S*. Typhimurium, clearly showed a larger but later transcriptional response to *S*. Choleraesuis. Both microarray and QPCR data provided evidence of a strong NF*κ*B-dependent host transcriptional response during *S*. Choleraesuis infection. Motif searches of orthologous human promoter sequences using TFM-Explorer and Clover software identified 51 putative novel NF*κ*B direct targets in the Early group (8–24 hpi) of genes and 145 in the Late group (48 hpi). While experimental validation is needed to confirm these predicted NF*κ*B binding sites and regulatory relationships, our study adds new data towards an understanding of the NF*κ*B signaling pathway response to Gram-negative bacterial infection which may be applicable across mammalian species.

## Methods

### Experimental design

Animals which were used in this study and experimental design were described previously [8]. Briefly, fifteen piglets which were confirmed to be fecal-negative for *Salmonella *spp. prior to challenge, were randomly allocated to the non-infected group (3 piglets) or to the infected group (12 piglets), respectively. The three non-infected control pigs were necropsied 3 days prior to experimental infection. On day 0, pigs in the infected groups were intranasally challenged with 1 × 10^9 ^CFU of *S*. Choleraesuis *χ*3246. Three randomly chosen infected pigs were necropsied at each time point of 8 hpi, 24 hpi, 48 hpi and 21 dpi, respectively. Tissue samples from the MLN were collected and immediately frozen in liquid nitrogen.

### Microarray hybridizations and data analysis

Five *μ*g total RNA was used for first and second strand cDNA synthesis according to the manufacturer instructions (Affymetrix, Inc. Santa Clara, CA). The double stranded cDNA was purified, tested on an Agilent Bioanalyser 2100, and served as the template for the subsequent *in vitro *transcription (IVT) reaction for cRNA amplification. Labeling cRNA with biotin was performed by the GeneChip^® ^One-Cycle target labeling kit (Affymetrix; Expression Analysis Technical Manual). Quality of the labeled cRNA was tested on an Agilent Bioanalyser 2100. Subsequently, labeled cRNA was fractionated and hybridized with the GeneChip^® ^Porcine Genome Array according to the standard procedures provided by the manufacturer. Chips were washed and stained with a GeneChip Fluidics Station 450 (Affymetrix, Inc. Santa Clara, CA) using the standard fluidics protocol. Chips were then scanned with an Affymetrix GeneChip Scanner 3000 (Affymetrix, Inc. Santa Clara, CA).

MAS 5.0 (Microarray Analysis System 5.0, Affymetrix, Inc. Santa Clara, CA) default normalization methods were used to obtain the expression measure for each probeset. Base-2 logarithms were then taken on these expression measures. The median of the log expression measures for each chip was then subtracted from all log expression measures on the same chip. Differentially expressed genes were identified by analyzing these normalized data with a linear model using SAS Proc GLM (SAS Institute, Cary, NC) on a gene by gene basis. The statistical model for gene *g *was as follows:, *y*_*ijg *_= *μ*_*g *_+ *T*_*ig *_+ *ε*_*ijg *_where *y*_*ijg *_is the log of the normalized signal for gene *g *on pig *j *necropsied at time *i*, *μ*_*g *_is an intercept term for gene *g*, *T*_*ig *_is the fixed effect of the *i*^th ^time-point on expression of gene *g*, and the *ε*_*ijg *_values are normally distributed random errors with mean 0 and gene-specific variances $\sigma_{g}^{2}$ > 0, assumed to be independent for each *g*. An *F *test for differences in expression across all time points during infection and *t*-tests for all ten pair-wise comparisons among the five treatment groups (non-infected, 8 hpi, 24 hpi, 48 hpi, and 21 dpi) were conducted as part of the analysis for each gene. This yielded eleven sets of *p*-values for the effect of infection. Each set of *p*-values was converted to a set of *q*-values using the method of Storey and Tibshirani (2003) [44]. The largest *q*-value in a list of genes declared to be differentially expressed provides an estimate of the upper bound of the positive False Discovery Rate (pFDR) associated with the list. Fold changes (FC) for each of the 10 pairwise comparisons were estimated as $2^{\left|{\hat{d}}_{kg}\right|}$, where ${\hat{d}}_{kg}$ is the estimated difference in means for gene *g *on the log scale for comparison *k*, *k *= 1, 2,⋯, 10. As the *p*-values tend to be stochastically smaller for genes with larger estimated fold changes in our data, we still use the largest *q*-value in a list of genes as a conservative estimate of the upper bound of pFDR even though we have excluded the genes with estimated FC < 2 (or estimated FC < 10 in some cases as noted in the Results section). Our microarray data have been submitted to the NCBI GEO database and the accession number is GSE7314.

### Transcriptome determination and Sequence-based annotation of Probesets

The transcriptome of normal and *S*. Choleraesuis infected MLN were determined as described previously [12]. Briefly, transcripts which showed a Present call for all three non-infected animals were counted in the transcriptome of normal porcine MLN tissue, while transcripts which showed a Present call for all three replicates in at least one time point during infection were counted as the transcriptome of infected porcine MLN tissue.

Probesets were annotated using novel consensus sequences produced from alignments from an assembly of approximately 1.7 million, publicity available porcine EST and full-length mRNAs, as follows. First, a blast analysis of these ISU consensus sequences against a well-curated sequence database, NCBI's RefSeq, was performed to obtain homologue and annotation information (Couture et al., unpublished observations). A relatively conservative cutoff of 1e-10 for an E-value was used for annotation of each consensus sequence. Second, the Affymetrix consensus sequences were blasted to the new ISU consensus sequences to annotate probesets designed from each Affymetrix consensus sequence. This assembly increased the length of available consensus sequences, which in turn produced higher scores to cross-species homologues than when the Affymetrix consensus sequences alone were used. Our E value cutoff created a minimum bit score of 74 which is more stringent that the minimum bit score of 50 which was used by Tsai and co-workers [45]. The current annotation of the entire Affymetrix Genechip is available upon request.

### Hierarchical Cluster analysis

After removing duplicate probe sets, a total of 1,853 genes showed *p*-value < 0.01 and estimated FC > 2 (*q*-value < 0.26) in at least one of the 10 possible time point pair-wise comparisons (8 h-C, 24 h-C, 48 h-C, 21 d-C, 24 h–8 h, 48 h–8 h, 21 d–8 h, 48 h–24 h, 21 d–24 h and 21 d–48 h) during *S*. Choleraesuis infection and were designated as the differentially expressed genes. This list was used to perform a hierarchical cluster analysis and to construct a heat map using the Gene Cluster 3.0 and tree view software (Stanford University, 2002).

### GO-slim creation and GO annotation of Affymetrix probesets

A set of high level GO terms which represent host response categories in biological_ process was selected by using OBO-Edit, which is part of the go-dev software provided by GO at Sourceforge . An expanded GO slim was created and GO analysis was performed for transcriptome and differentially expressed genes as described before [12]. Fisher's exact test was used for gene enrichment analysis.

### Quantitative PCR (QPCR) RNA analysis

Quantitative PCR technology was used to verify the differential expression of 21 genes at early response stages (8 hpi, 24 hpi and 48 hpi), as identified by the microarray. The TGM3 gene, which has not yet been annotated on the microarray, was also analyzed. The RPL32 gene, a reference gene for high abundance gene transcripts, was used as a positive control. All probes and primers for real time TaqMan PCR were designed as previously described [26,46]. The interpolated number (C_t_) of cycles to reach a fixed threshold above background noise was used to quantify amplification. The fold change in expression of the target gene was estimated as 2^ΔCt^, where ΔC_t _is the difference between average C_t _values for the control and infected pigs. Resulting Q-PCR data were analyzed by one-way ANOVA on a gene by gene basis, as done in analyzing microarray data, but using JMP 5.0 Software (SAS Inc, Cary, NC). Fisher's LSD post-hoc test was applied to assess differences between groups of pigs at different time points post infection. A value of *p *≤ 0.05 was considered statistically significant.

### NF*κ*B motif searching

TFM-Explorer identifies windows conserved amongst a group of sequences sharing a common transcription factor binding site. To do this, TFM-Explorer compares the input sequences to a set of previously derived position specific scoring matrices (PSSM) for the binding sites of interest to obtain a score for each sequence. It then compares this to the probability of the binding site appearing at random in the genomic background sequences. It then sees how many of the sequences have the same matrix within a window from 300 to 1500 bases long. The closer the sequence matches the PSSM, and the higher the percentage of sequences with the binding site within a window, the more significant that window becomes. The default parameters of TFM-Explorer, including the limit of 500 input sequences, were used for all runs.

The 1,500 bp 5' and 500 3' bp (relative to the annotated transcription start site) of the human orthologs for all annotated and upregulated DE porcine genes were obtained using PERL scripts. These scripts use human RNA RefSeq accession number, obtained from BLAST results using the Affymetrix porcine consensus sequence (Couture et al., unpublished results) that identified orthologs, to identify and download human chromosome GenBank and FASTA files from NCBI RefSeq. Information on transcription start sites from the human chromosomal GenBank files, strand information, and sub-sequences for each transcript were extracted from the FASTA files. Human orthologs of the porcine differentially expressed genes were then separated into different groups: two early sets (one of 83 sequences with known and unknown NF*κ*B targets [E83] and one of 61 sequences without these known targets [E61]); two late sets (one of 319 sequences with known NF*κ*B targets [L319] and one of 283 sequences without these known targets [L283]); five different randomly selected sets of 500 (A500) from the 560 up-regulated and annotated genes across all time points (due to the limitation of TFM-Explorer of allowing a maximum of 500 input sequences); and a set of 475 genes from this 560 gene set with the known targets of NF*κ*B removed (U475). A list of genes known to be direct targets of NF*κ*B was obtained at two websites:  and . The promoter sequences in these different groups were then searched for windows of sequence with over-representation of any of the available known NF*κ*B motifs defined by position-specific sequence matrices deposited in public TRANSFAC 7.0  or JASPAR  using the default parameters of TFM-Explorer [18].

In addition to TFM-Explorer, Clover [19] was also used to find putative binding sites in the above promoter sequences for E83, L319, and for all 560 differentially expressed genes. Clover can use a permutation test to see how often a given matrix is found in a group of sequences when compared to how often it is found in a randomly generated sequence set selected from background sequences that maintain the same number of sequences, the same length, and same G/C content as the input set. Since human sequences were used as input, sets of human sequence was used as background sequence as well; human CpG islands, the entire chromosome 20, and the 2,000 upstream bases from genes. Only the default number of permutation tests was changed: using 5,000 instead of the default of 1,000. Also, since Clover does not have an input size limit all 560 sequences were run as a single group. After running both programs on the same set of sequences, the intercept of the sequences was defined to be putative NF*κ*B targets.

## Authors' contributions

YFW participated in the Microarray interpretation work, including data organization, cluster analysis, pathway analysis, Q-PCR work, and drafted the manuscript. OPC performed the NF*κ*B promoter analysis and created the GO-slim tool, and contributed to the manuscript. QL, JCMD and DN carried out microarray data analyses, and LQ contributed to the manuscript. JJU and SMDB participated in the animal work, tissue collection, RNA preparation. DK and JKL participated in Q-PCR and data analysis. CKT, SMDB and JKL conceived the study, participated in the study design, supervised the overall process and revised the manuscript. All authors have read and approved the final manuscript.

## Supplementary Material

Additional file 1 Transcriptome analysis of porcine MLN during *S*. Choleraesuis infection. Probesets that showed a present call for all three non-infected animals were added to the probsets which showed a present call for all three replicates in at least one time point during infection. Thus 16, 046 probesets were counted as the transcriptome (detected expression in at least one condition) in porcine mesenteric lymph nodes. Click here for file

Additional file 2 Differentially expressed gene list during *S*. Choleraesuis infection. 1,853 genes that showed differentially expressed with statistical significance in at least one of all 10 pair-wise comparisons during infection (p < 0.01, fc > 2). Click here for file

Additional file 3 Upregulated known NFkB target genes during *S*. Choleraesuis infection. 58 known NF*κ*B target genes were induced significantly at 8, 24 and/or 48 hpi. These target genes can be classified into two groups based on their expression pattern; a "Early group" in which genes were induced at either 8 or 24 hpi, and a "Late group" in which genes were only induced at 48 hpi. Click here for file

Additional file 4 Putative and known NFkB target genes in "early", "late" and "all" groups. All genes in the defined "early", "late" and "all" groups are shown, along with a) for which of these genes both Clover and TFM Explorer predicted an NF*κ*B site was present in the 2,000 bp orthologous human promoter sequence (-1,500 to +500 bp relative to the transcription start site annotated at NCBI); and b) which genes are known to be NF*κ*B targets genes using the websites shown in Additional file 3. Click here for file
